# Progression of *GBA1* severe and risk variants: a longitudinal mixed model analysis

**DOI:** 10.3389/fnagi.2026.1771789

**Published:** 2026-03-26

**Authors:** Anne-Marie Hanff, Christopher McCrum, Armin Rauschenberger, Sinthuja Pachchek, Gloria A. Aguayo, Claire Pauly, Sonja R. Jónsdóttir, Olena Tsurkalenko, Laure Pauly, Zied Landoulsi, Anja K. Leist, Patrick May, Maurice P. Zeegers, Rejko Krüger

**Affiliations:** 1 Faculty of Sciences Technology and Medicine, University of Luxembourg, Esch-sur-Alzette, Luxembourg; 2 Transversal Translational Medicine, Luxembourg Institute of Health, Strassen, Luxembourg; 3 Department of Epidemiology, CAPHRI Care and Public Health Research Institute, Maastricht University Medical Centre+, Maastricht, Netherlands; 4 Department of Nutrition and Movement Sciences, NUTRIM School of Nutrition and Translational Research in Metabolism, Maastricht University Medical Centre+, Maastricht, Netherlands; 5 Bioinformatics & AI, Department of Medical Informatics, Luxembourg Institute of Health, Strassen, Luxembourg; 6 Translational Neurosciences, Luxembourg Centre for Systems Biomedicine, University of Luxembourg, Esch-sur-Alzette, Luxembourg; 7 Department of Precision Health, Luxembourg Institute of Health, Strassen, Luxembourg; 8 Parkinson Research Clinic, Centre Hospitalier de Luxembourg, Luxembourg, Luxembourg; 9 Digital Medicine Group, Luxembourg Institute of Health, Strassen, Luxembourg; 10 Department of Social Sciences, University of Luxembourg, Esch-sur-Alzette, Luxembourg; 11 Department of Epidemiology, Care and Public Health Research Institute, School of Nutrition and Translational Research in Metabolism, Maastricht University, Maastricht, Netherlands

**Keywords:** clinical deterioration, epidemiology, Gaucher disease, human genetics, Parkinson disease, precision medicine

## Abstract

**Introduction:**

An association between severe *GBA1* variants and the progression of non-motor symptoms in PD has been reported, but the role of Parkinson’s-risk (PD-risk) *GBA1* variants is less clear.

**Methods:**

We assessed symptom progression in individuals with severe and PD-risk variants compared to non-carriers. We analyzed longitudinal data from 726 individuals with typical PD, including 22 carriers of severe *GBA1* variants and 47 carriers of PD-risk *GBA1* variants.

**Results:**

The findings were not significant after adjusting for Bonferroni correction; however, linear mixed models analyses showed that at a nominal significance level of 5%, carriers of PD-risk or severe variants were associated with faster cognitive decline compared to non-carriers. Moreover, carriers of PD-risk variants were associated with faster worsening of apathy, quality of sleep, tremor, and non-motor symptoms [Movement Disorder Society-Unified Parkinson’s Disease Rating Scale (MDS-UPDRS I)] compared to non-carriers; however, we did not observe this tendency in individuals with severe variants.

**Discussion:**

The exploratory study suggests associations between PD-risk variants and a more rapid disease progression among carriers compared to non-carriers. Nevertheless, the findings should be interpreted cautiously and require confirmation in an independent cohort before any reevaluation of their pathologic relevance.

## 1 Introduction

The development of Parkinson’s disease (PD) is primarily influenced by genetic and environmental factors (Simon et al., 2020). Variants in the glucocerebrosidase (*GBA1*) gene (*GBA1* [OMIM 606463]) can cause Gaucher’s disease, a recessive lysosomal storage disorder, and result in reduced activity of the lysosomal enzyme glucocerebrosidase (GCase). This reduced activity, in turn, is linked to increased alpha-synuclein aggregation, which is involved in the pathogenesis of PD (Mazzulli et al., 2011; Sidransky and Lopez, 2012). While an association between these severe *GBA1* variants and the progression of non-motor symptoms in PD has been reported (Gan-Or et al., 2015), the role of PD-risk *GBA1* variants is less clear (Goldstein et al., 2019; Menozzi and Schapira, 2021; Petrucci et al., 2020).

Based on the resulting PD severity, amino acid changes in the severe *GBA1* variants can be classified as severe or mild, while amino acid changes in the PD-risk *GBA1* variants can be considered higher risk for PD (Hoglinger et al., 2022). Previous research has shown that individuals carrying severe *GBA1* variants experience an earlier onset and more severe motor, cognitive, olfactory, and psychiatric symptoms (Goldstein et al., 2019; Liu et al., 2016; Thaler et al., 2018). However, the disease progression of individuals with PD who carry the *GBA1* variant p.N409S, which is considered mild, remains unclear (Cilia et al., 2016; Menozzi and Schapira, 2021; Petrucci et al., 2020). In the Luxembourg Parkinson study, the PD-risk variant p.E365K is the most prevalent *GBA1* variant among individuals with PD (Pachchek et al., 2023). Moreover, these variants that were considered PD-risk are associated with a higher risk for cognitive impairment (Straniero et al., 2020) and a lower risk for motor deterioration (Maple-Grodem et al., 2021) compared to non-carriers (Koros et al., 2022).

These findings emphasize the complexity of the relationship between *GBA1* and PD and highlight the need for a better understanding of the mechanisms by which these variants contribute to PD (Goldstein et al., 2019). Therefore, a detailed description of longitudinal changes in both motor and non-motor symptoms among carriers of severe *GBA1* variants and PD-risk *GBA1* variants, compared to non-carriers, could elucidate their pathogenic relevance. Moreover, such comprehensive investigations of both motor and non-motor symptoms could help to estimate effect sizes for designing clinical trials for disease-modifying therapies. Therefore, we aimed to provide an overview of symptom progression in individuals with PD. We describe the progression of motor and non-motor symptoms among individuals with PD carrying heterozygous, Gaucher-related severe and PD-risk *GBA1* variants compared to non-carriers and quantify the moderating effect of *GBA1* variants using a large, single-center longitudinal cohort.

## 2 Materials and methods

### 2.1 Study design, setting, participants, and study size

This retrospective analysis is part of the Luxembourg Parkinson’s study, a nationwide, single-center, observational, longitudinal-prospective, and dynamic cohort (Hipp et al., 2018; Pavelka et al., 2023). The completed STROBE (Vandenbroucke et al., 2007) reporting guideline checklists are provided in Supplementary Material 4. Our analysis includes participants diagnosed by a neurologist within the framework of the Luxembourg Parkinson’s study with typical PD or PD with dementia (PDD) based on the United Kingdom PD Society Brain Bank Clinical Diagnostic Criteria (Hughes et al., 1992). Participants resided either at home or in nursing homes within Luxembourg and the Greater Region (geographically proximate areas). Recruitment started in 2015, with subsequent annual follow-ups. The primary objective of the Luxembourg Parkinson’s Study was to facilitate stratification and differential diagnosis of PD (Hipp et al., 2018; Pavelka et al., 2023).

### 2.2 Variables, data sources, and measurement

The outcomes of interest were the progression (i.e., change per additional year since diagnosis) of 15 motor and non-motor symptoms. Table 1 provides detailed information regarding the characteristics of these outcomes, their data sources, and the assessment methods. All outcomes were numerical and assessed during annual follow-ups, with variations of up to 3 months to minimize seasonal effects. As individuals with PD were enrolled at different time points (Twisk, 2013) due to the dynamic cohort study design, the disease progression could be distinguished from cohort or period effects. Only individuals with PD who had data for time since diagnosis were included in the longitudinal analysis. We described differences in demographic and health-related characteristics at baseline between the three groups instead of controlling for confounders. We only included time to diagnosis (years from the first motor symptoms to the diagnosis) to correct for delayed reporting of years since diagnosis.

**Table 1:** Instrument, assessment types, and variable names of the included constructs.

Construct intended to measure	Instrument	Assessment type	Details
Patient-reported outcomes			
Apathy	SAS (Starkstein et al., 1992)	PROM	Numerical score (0–42)
Depression	BDI-I (Beck et al., 1988)	PROM	Numerical score (0–63)
Dysphagia	MDT-PD (Buhmann et al., 2019; Simons et al., 2019)	PROM	Numerical score (3–103)
Functional mobility	FMCS (Hanff et al., 2023)	PROM	Numerical score (0–100)
Non-motor symptoms	MDS-UPDRS I (Martinez-Martin et al., 2013)	Patient-Reported and Clinician-Assessed Outcome Measure	Numerical score (0–52)
Motor symptoms	MDS-UPDRS II (Martinez-Martin et al., 2013)	PROM	Numerical score (0–52)
Pain	PDQ-39 subscale bodily discomfort (Peto et al., 1995)	PROM	Numerical score (0–100)
Quality of sleep	PDSS (Chaudhuri et al., 2002)	PROM	Numerical score (0–150)
Rem-sleep behavior disorders	RBDSQ (Stiasny-Kolster et al., 2007)	PROM	Numerical score (0–13)
Clinician-assessed outcomes or performance tests			
Global cognition	MoCA Total Score (Nasreddine et al., 2005)	Performance test	Numerical score (0–30)
Motor symptoms	MDS-UPDRS III (Martinez-Martin et al., 2013)	Clinician-Assessed Outcome Measure	Numerical score (0–132)
Motor fluctuations	MDS-UPDRS IV (Martinez-Martin et al., 2013)	Clinician-Assessed Outcome Measure	Numerical score (0–24)
Olfaction	ODOFIN Sniffin’ Sticks Identification Test 16	Performance test	Numerical score (0–16)
Postural instability and gait disorder	PIGD score (Jankovic et al., 1990; Stebbins et al., 2013)	Patient-Reported and Clinician Assessed Outcome Measure	Numerical score (0–20)
Tremor	Tremor scale (Forjaz et al., 2015; Jankovic et al., 1990)	Patient-Reported and Clinician Assessed Outcome Measure	Numerical score (0–4)
Exposure			
Time variant with baseline assessment and yearly follow-up	Time since diagnosis (y.): Date of assessment—Date of diagnosis	Interview	Numerical value
Covariates			
Time variant with baseline assessment and yearly follow-up	Time to diagnosis (y.): Date of diagnosis—Date of first motor symptoms	Interview	Numerical value
Moderators			
*GBA1* variants	Name of the amino-acid changes	Genotyping	Variable with 13 categories
No of carriers of *GBA1* variants in individuals with PD	Classification by Hoglinger et al. (2022)	Genotyping	Variable with 4 categories

PROM, BDI-I, Beck Depression Inventory I, FMCS, Functional Mobility Composite Score, MDS, Movement Disorders Society, MDT, Munich Dysphagia Test, MoCA, Montreal Cognitive Assessment, PDQ39, Parkinson’s Disease Questionnaire, PDSS, Parkinson’s Disease Sleep Scale, PIGD, Postural Instabilities and Gait Disorders, RBDSQ, RBD Screening Questionnaire, SAS, Starkstein Apathy Scale, UPDRS, Unified Parkinson’s Disease Rating Scale.

DNA was extracted from peripheral blood samples. The samples underwent genotyping using the NeuroChip (Blauwendraat et al., 2017) and additional long-read PacBio sequencing, targeting the *GBA1* locus (Pachchek et al., 2023). Variants in known PD-related genes were validated by Sanger sequencing for single-nucleotide variants or Multiplex Ligation-dependent Probe Amplification (MLPA) for copy number variants (CNVs). Individuals who were carriers of variants in other PD-related genes were excluded (Landoulsi et al., 2023; Pachchek et al., 2023). All *GBA1* variants were annotated based on the GRCh37 assembly and numbered according to current HGVS guidelines.^1^ We used the primary translation product (NM_001005742.3), which includes the 39-residue signal peptide, as the reference sequence. While we have used this standardized nomenclature throughout the article for genomic accuracy, the corresponding “legacy” names (e.g., p.L444P for p.L483P) are provided in Supplementary Table S1 in Supplementary Material 1 to facilitate comparison with earlier literature, as these remain widely referenced in the clinical community. Supplementary Table S1 in Supplementary Material 1 also shows the genotypes and amino acid changes for all individuals. Among the 76 carriers of the most prevalent variant p.E365K, 2 were homozygous and 74 were heterozygous. Consequently, under the assumption of a dominant model, we combined the heterozygous and homozygous carriers. In total, we found 12 *GBA1* variants (PD-risk or severe variants, as listed in Table 1).

Variants in the *GBA1* gene were classified according to their established impact on both GD and PD risk, following the framework by Hoglinger et al. (2022). Specifically, variants were grouped into three categories: severe variants, which cause severe GD phenotypes and carry the highest risk for PD (e.g., p.L483P); mild variants, which cause milder GD phenotypes with moderate risk of PD (e.g., p.N409S); and risk variants, which are non-pathogenic for GD but significantly increase susceptibility to PD (e.g., p.E365K). While all identified *GBA1* variants function as PD risk factors due to incomplete penetrance, this nomenclature distinguishes them according to their clinical severity in GD and their specific odds ratios for PD. Due to a limited number, the mild variants were excluded from this analysis. The other participants were considered non-carriers of *GBA1* variants. Supplementary Table S2 describes the different variants and their classification by involvement in PD. Exonic or splice-site variants that are not mentioned in this article were subclassified as severe *GBA1* variants if they were annotated as pathogenic in the archive of reports of relationships among medically important variants and phenotypes (ClinVar, RRID:SCR_006169; Landrum et al., 2014). In two cases, two nucleotide–protein changes co-existed. Those indicated with an “a or b” in Supplementary Table S2 were classified as severe variants (Hoglinger et al., 2022). Further details on genotyping, *GBA1* variant annotation and validation, along with details on nomenclature and classification, can be found in a study describing the original *GBA1* work (Landoulsi et al., 2023; Pachchek et al., 2023).

### 2.3 Statistical methods

Data analysis was performed using R, version 4.3.2 (R Core Team, 2021). To assess whether a carrier status for *GBA1* variants (classified as PD-risk or severe) was associated with a different effect of time since diagnosis on motor and non-motor symptoms, we created one interaction model per outcome. In each model, a three-group categorical variable representing *GBA1* status (PD-risk, severe, and non-carriers [reference group]) was included, along with its interaction with time since diagnosis.

Consequently, we performed longitudinal two-level mixed model analyses using the “lmer” function from the “lme4” package (Bates et al., 2015). Models were estimated using the maximum likelihood method, with years since diagnosis as a fixed effect, along with a random intercept and a random slope at the participant level. After adding the random intercept at the subject-level,^2^ we assessed the need tp include a random slope for time by performing a likelihood ratio test^3^ (using the “anova” function of the “lme4”-package (Bates et al., 2015), method = “lrt”) to compare the model with^4^ and without^1^ a random slope for time (i.e., years since diagnosis). Finally, in addition to the linear fixed effect for time, we tested a quadratic and a cubic function. We followed the hierarchy of effects by including main effects when testing interaction effects. The estimates and their 95% confidence intervals (CI) for the interaction effect between time since diagnosis and the different *GBA1* variants describe the additional annual change that occurred in the group of interest in the 15 outcomes relative to the reference group of non-carriers. The modification of the effect of time since diagnosis on an outcome by the different *GBA1* variants was evaluated by the statistical significance of the interaction term (t-test) at a Bonferroni-adjusted significance level (alpha = 0.05/(15 outcomes*3 variants) = 0.001). Statistical significance and confidence intervals for the mixed models were obtained using the Kenward–Roger approximation for degrees of freedom. We emphasized the estimates and the uncertainty by explicitly discussing the lower and upper 95% confidence intervals. Thus, all *p*-values, independent of the statistical significance, will be reported (Amrhein et al., 2019). We calculated estimated marginal means using the “ggpredict” function of the “ggeffects” package (Lüdecke, 2018), summarized the interaction coefficients, and illustrated the interactions using the “plot_model” function of the sjPlot package (Lüdecke, 2025).

## 3 Results

Table 2 summarizes key study characteristics to help understand the potential applicability and, thus, the generalizability of the findings. As illustrated in the flowchart (Supplementary Figure S1) in Supplementary Material 2, until the date of data export (2024-01-31), 990 individuals with Parkinsonism participated in the Luxembourg Parkinson’s Study. After excluding individuals with atypical PD, those without genetic testing, or those with other pathogenic PD-related variants, we included 726 individuals with typical PD who had a baseline assessment between 2015-03-04 and 2024-01-29. Supplementary Table S3 in Supplementary Material 1 provides a description of the 726 study participants and missing data. A total of 488 individuals with typical PD (66.6%) were men. In the overall cohort at the first assessment, the median age was 68.2 years (IQR 14.5 years), and the median time since diagnosis was 3.2 years (IQR 6.3 years). The average number of visits per patient was 3.0 (IQR 3.0), ranging from 1 to 8, and 406 patients (55.4%) had 3 or more follow-up visits. The median MDS-UPDRS III score was 32.0 (IQR 22.0), and the median Hoehn & Yahr stage was 2.0 (IQR 0.5).

**Table 2:** Key characteristics.

Sample size	726	
Data collection period	2015-03-04 to 2024-01-29	
Study design	Cohort	
Setting	Individuals with typical PD living at home or in a nursing home in Luxembourg and the Greater Region	
Inclusion criteria	Individuals with typical PD and PDD	
Outcomes Concept (Measure)	Apathy (SAS), depression (BDI-I), functional mobility (FMCS), LEDD (mg/kg), non-motor symptoms (MDS-UPDRS I), patient-reported motor symptoms (MDS-UPDRS II), clinician-assessed motor symptoms (MDS-UPDRS III), motor complications (MDS-UPDRS IV), dysphagia (MDT-PD), global cognition (MoCA), olfaction (Sniffin’ Sticks), bodily discomfort (PDQ-39 subscale bodily discomfort), health-related quality of life (PDQ-39), quality of sleep (PDSS), postural instabilities and gait disturbances (MDS-based PIGD), REM sleep behavior disorder (RBDSQ), tremor (MDS-based tremor scale)	
Sex	488 (66.6%) male 245 (33.4%) female	
Age	68.2 (IQR 14.5)	
Disease stage	2.0 (IQR 0.5)	
No. of carriers of *GBA1* variants stratified by involvement in PD	No *GBA1* variant	657 (89.6%)
	PD-Risk p.E365K, p.T408M	47 (6.4%)
	Severe p.F252I, p.G234W, p.G241R, p.G416S, p.L483P, p.P161S, p.R398X, p.R502H, c.115 + 1G > A	22 (3.0%)
Determinants	Time since diagnosis, time to diagnosis	

Categorical variables: counts (%), numerical variables: Median (IQR). BDI-I, Beck Depression Inventory I, FMCS, Functional Mobility Composite Score, MDS, Movement Disorders Society, MDT, Munich Dysphagia Test, MoCA, Montreal Cognitive Assessment, PDQ39, Parkinson’s Disease Questionnaire, PDSS, Parkinson’s Disease Sleep Scale, PIGD, Postural Instabilities and Gait Disorders, RBDSQ, RBD Screening Questionnaire, SAS, Starkstein Apathy Scale, UPDRS, Unified Parkinson’s Disease Rating Scale.

Table 3 describes the baseline characteristics of non-carriers and carriers of variants considered PD-risk or severe. We included 22 heterozygous carriers of severe *GBA1* variants and 47 heterozygous carriers of PD-risk *GBA1* variants, while 657 individuals carried no *GBA1* variant. The carriers of severe variants had a longer time since diagnosis (7.2 years) compared to the non-carriers and carriers of PD-risk variants (3.7 years).

**Table 3:** Baseline characteristics of non-carriers and carriers of variants considered as PD-risk or severe.

Variables	Non-carriers (*N* = 657)	PD-risk variants (*N* = 47)	Severe variants (*N* = 22)
Sociodemographic characteristics			
Age (y)	68.2 (14.6)	68.8 (11.4)	63.9 (16.7)
Male sex	442 (64.2%)	28 (59.6%)	13 (59.1%)
Years of education	13.0 (6.0)	12.0 (4.0)	14.0 (6.2)
Health-related characteristics			
Time since diagnosis (y)	3.2 (6.2)	3.7 (4.4)	7.2 (7.6)
Age at diagnosis (y)	63.0 (17.0)	63.0 (11.5)	57.0 (19.0)
Age at onset of motor symptoms (y)	61.0 (17.5)	59.0 (11.8)	56.5 (22.0)
Time to diagnosis (y)	1.0 (3.0)	2.0 (4.0)	1.0 (2.0)
MDS-UPDRS I	9.0 (9.0)	8.0 (8.0)	16.0 (10.0)
MDS-UPDRS II	9.0 (11.0)	11.0 (9.5)	11.0 (7.0)
MDS-UPDRS III	32.0 (23.0)	28.5 (16.2)	32.0 (25.0)
MDS-UPDRS VI	0.0 (0.0)	0.0 (0.0)	0.0 (4.8)

Categorical variables: counts (%), numerical variables: median (IQR), Time to diagnosis = Date of diagnosis—Date of first motor symptoms, y. = years, PDD, PD Dementia.

While the majority of outcomes showed a linear trajectory, this was not the case for apathy (SAS), global cognition [Montreal Cognitive Assessment (MoCA)], bodily discomfort [Parkinson’s Disease Questionnaire-39 (PDQ-39) subscale bodily discomfort], patient-reported motor symptoms (MDS-UPDRS III), motor complications (MDS-UPDRS IV), and postural instability and gait disturbances (MDS-UPDRS-based PIGD score), for which adding the quadratic effect significantly improved the model fit.

**Figure 1:**
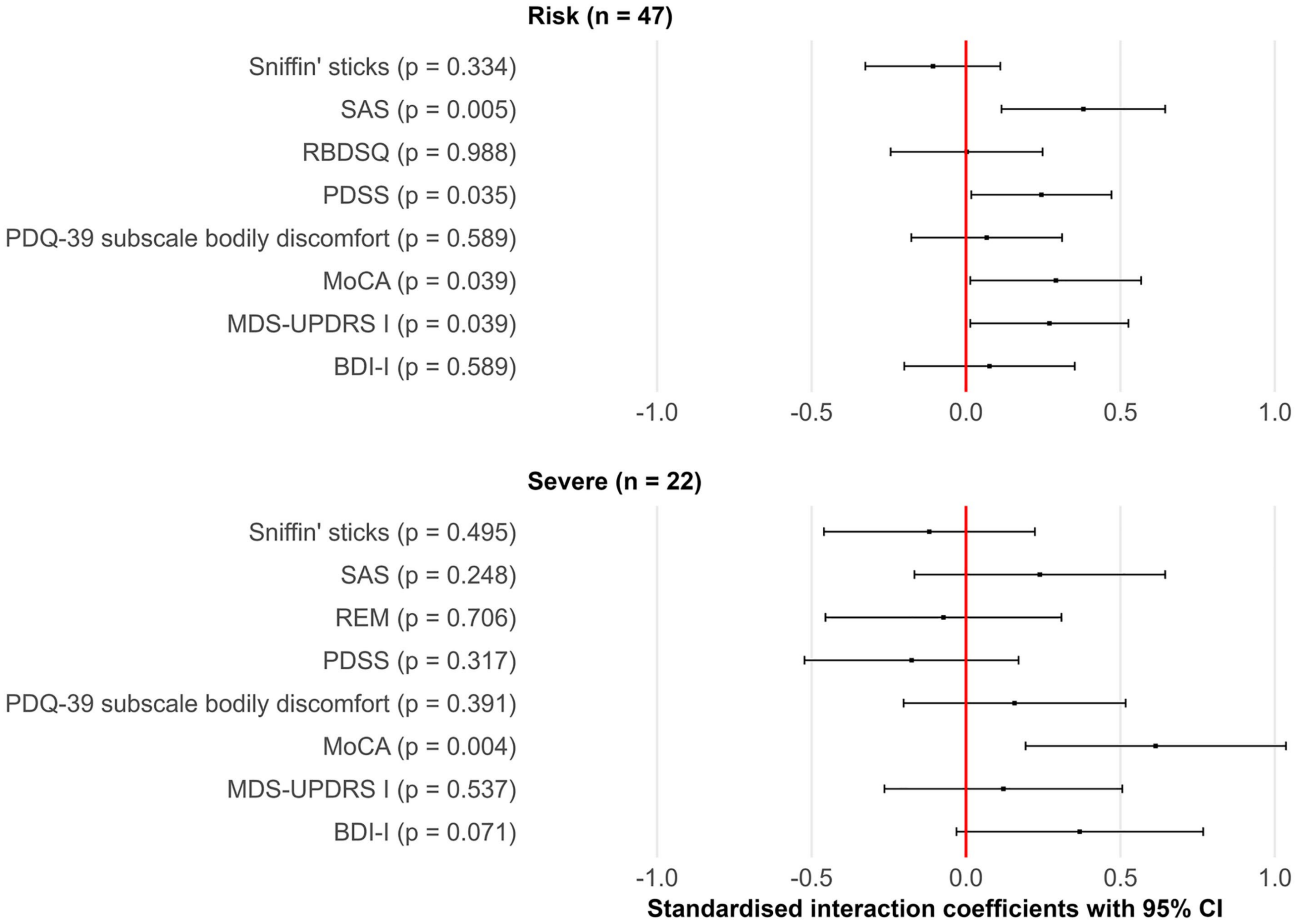
Association of variants considered PD-risk and severe variants with progression of non-motor symptoms (interaction coefficients). Right side of the red line = associated with worse progression. BDI-I, Beck Depression Inventory I; MDS, Movement Disorders Society; MoCA, Montreal Cognitive Assessment; PDQ-39, Parkinson’s Disease Questionnaire-39; PDSS, Parkinson’s Disease Sleep Scale; RBDSQ, RBD Screening Questionnaire; SAS, Starkstein Apathy Scale; UPDRS, Unified Parkinson’s Disease Rating Scale.

In Supplementary Material 3, we describe the association of the different variants with the progression of motor and non-motor symptoms. We illustrate the association between the different variants considered PD-risk or severe and the progression of non-motor symptoms in the forest plot in Figure 1, while we illustrate the association with motor symptoms in Supplementary Figure S2 in Supplementary Material 2. Although, after Bonferroni-adjusted significance levels (alpha = 0.05/(15 outcomes*2 variants) = 0.0017), the findings did not remain significant, at an unadjusted significance level (alpha = 0.05), 47 individuals carrying the PD-risk *GBA1* variants were associated with faster progression compared to non-carriers, specifically annual change since diagnosis, in apathy (SAS) (0.380, 95%CI: 0.115, 0.645, *p* = 0.005), global cognition (MoCA) (0.291, 95%CI: 0.014, 0.567, *p* = 0.039), quality of sleep (PDSS) (0.244, 95%CI: 0.017, 0.471, *p* = 0.035), tremor (MDS-UPDRS based tremor scale) (0.258, 95%CI: 0.001, 0.515, *p* = 0.050), and non-motor symptoms (MDS-UPDRS I) (0.270, 95%CI: 0.014, 0.526, *p* = 0.039). Finally, 22 individuals carrying the severe *GBA1* variants were associated only with a faster cognitive decline (MoCA) compared to non-carriers (0.614, 95%CI: 0.193, 1.036, *p* = 0.004).

For illustrative purposes in Figure 1 and Supplementary Figure S2, the following scores were inverted so that higher values indicate worse outcomes: functional mobility (FMCS), quality of sleep (PDSS), global cognition (MoCA), and olfaction (Sniffin’ Sticks).

## 4 Discussion

This study provides a comprehensive overview of the association between different types of *GBA1* variants and the progression of various symptoms in PD and explores the role of PD-risk variants on disease progression. The findings were not significant after the conservative Bonferroni-adjustment, which could partially be explained by the high number of outcomes. However, this is also a strength of our study because we use longitudinal data; therefore, we discuss the unadjusted significant results in the following paragraph. Thus, those results need to be interpreted with caution until a validation of our findings is performed in an independent cohort.

First, carrying PD-risk or severe *GBA1* variants was associated with faster cognitive decline compared to non-carriers. Second, carriers of PD-risk *GBA1* variants were associated with faster worsening of apathy, quality of sleep, tremor, and non-motor symptoms (MDS-UPDRS I) compared to non-carriers; however, this tendency was not observed in individuals with severe variants.

### 4.1 Association of different GBA1 variant types with progression

Our findings suggest that PD-risk *GBA1* variants are associated with a more rapid worsening of non-motor symptoms and tremor. Specifically, we observed that, before Bonferroni adjustment, both PD-risk and severe *GBA1* variants were associated with more pronounced cognitive decline. Additionally, carrying a PD-risk *GBA1* variant was associated with faster worsening of apathy, while this tendency was not observed in individuals with severe variants. Finally, although the confidence intervals were overlapping, the interaction effect of the *GBA1* variant and years since diagnosis on global cognition was stronger in individuals with variants considered severe (0.614, 95%CI: 0.193, 1.036, *p* = 0.004) compared to individuals with PD-risk variants (0.291, 95%CI: 0.014, 0.567, *p* = 0.039). Our results are consistent with previous research (Thaler et al., 2018), as we observed a non-significant tendency for faster progression of depression in individuals with severe variants, while this tendency was not observed in the more common PD-risk variants. The PD-risk *GBA1* variants were the only variants with evidence for an association with the progression of a motor symptom, specifically tremor.

### 4.2 Strengths and limitations

This study has several strengths and limitations. Notably, we enhanced the generalizability of our findings by analyzing data from all participants of the Luxembourg Parkinson’s Study, including individuals with PD or PDD from Luxembourg and the Greater Region, who were treated and lived in varied settings and environments. More specifically, the demographic range included individuals with PD of both sexes, speaking different languages, aged 32–93 years, with educational backgrounds ranging from 1 to 30 years, with years since diagnosis ranging from 0 to 32. A significant proportion (68.3%) of the individuals with PD were in disease stages H&Y 1–2, with overall disease stages ranging from H&Y 1 to H&Y 5.

In terms of methodology, we used advanced statistical techniques to estimate changes over time in our longitudinal dataset. By using mixed models, we accounted for intra-individual changes and provided a comprehensive description of symptom progression in carriers of different *GBA1* variants compared to non-carriers. Our study had some limitations, including potential reasons for missing data, such as disease progression, the COVID-19 pandemic, and deaths since baseline assessment. Additionally, higher rates of missing values were observed for the Munich Dysphagia Test (MDT) score, likely due to its later inclusion in the study. Therefore, the analyses of this outcome should be considered exploratory. Despite the potential sampling bias for the analyses involving the MDS-UPDRS III on-site test, the frequency of missing data at follow-up was similar in carriers and non-carriers. We assumed data were missing at random (MAR), which can be handled by mixed models without requiring imputation (Twisk et al., 2013). To further minimize missing data and information bias, we established standardized data collection procedures. Questionnaires were sent to the patients prior to their visit, allowing them to complete them at home at their convenience. If a participant could not attend follow-up visits, either at the center or via the mobile recruitment team, we offered a standardized telephone questionnaire with a reduced assessment.

As the classification of *GBA1* variants, in particular those of unknown significance, is still under discussion, and as the number of different variant types considered PD-risk or severe is still limited, our results provide hypotheses for future, larger research projects, such as the monogenic GP-2 project (Lange et al., 2023).

Our research described the progression since the diagnosis. Future research should use data from risk and prodromal cohorts to describe the biological progression before the diagnosis of PD (Chahine et al., 2023). Therefore, as we focused on the progression of clinical symptoms, future research should also evaluate the biological progression by analyzing a larger sample of individuals with variants considered PD-risk or severe *GBA1* variants, with follow-up starting from the detection of an abnormal α-synuclein seed amplification assay (SAA), because the disease has already biologically progressed before clinical symptoms manifest (Chahine et al., 2023). As we used the MoCA score, a tool primarily developed and validated to screen for Mild Cognitive Impairment (Nasreddine et al., 2005), future research should measure cognitive decline with a longitudinal, detailed cognitive assessment including visuo-spatial functions to further differentiate the progression of diverse cognitive sub-domains in individuals with and without *GBA1* variants (Afshari et al., 2025; Federoff et al., 2012; Kaivola et al., 2022; Koros et al., 2022; Mengel et al., 2016; Pavelka et al., 2022; Pillai et al., 2021).

In conclusion, our study provides a comprehensive overview of the association between different *GBA1* variant types and the progression of motor and non-motor symptoms based on longitudinal data. The detailed figures illustrating the progression should facilitate the interpretation of the symptoms’ trajectories in individuals with the different *GBA1* variants by health professionals. Our study helps to clarify the association of the PD-risk *GBA1* variants with disease progression, and our results highlight the importance of including PD-risk variants in comprehensive research projects, as we could not confirm previous findings (Huh et al., 2020; Omer et al., 2022; Thaler et al., 2018) reporting that non-motor symptoms progress primarily in individuals with severe *GBA1* variants. As the progression of severe *GBA1* variants appears to be different, we recommend that they be studied separately (Thaler et al., 2018). Future research combining data across cohorts should test our nominally significant findings to further elucidate the pathogenic relevance of variants considered PD-risk or severe *GBA1* variants in men and women with Parkinson’s disease, increase statistical power, and provide more definitive conclusions.

## Data Availability

The datasets presented in this article are not readily available due to privacy restrictions and concerns regarding participant consent surrounding data dissemination. Requests for data access can be submitted to the NCER‑PD Data and Sample Access Committee at request.ncer-pd@uni.lu, where they will be reviewed according to the governance framework validated by national authorities.

## Footnotes

1
http://varnomen.hgvs.org

2 model_1 < − lme4:lmer(outcome~disease_duration+(1|ND), REML = FALSE, data = reduced_data).

3 anova(model_1, model_2).

4 model_2 < − lme4:lmer(outcome~disease_duration+(1 + disease_duration|ND), REML = FALSE, data = reduced_data).

## Group member of NCER-PD Consortium

Luxembourg Parkinson’s study as listed below: Geeta Acharya, Luxembourg Institute of Health, Strassen, Luxembourg; Gloria Aguayo, Luxembourg Institute of Health, Strassen, Luxembourg; Myriam Alexandre, Luxembourg Institute of Health, Strassen, Luxembourg; Muhammad Ali, Luxembourg Centre for Systems Biomedicine, University of Luxembourg, Esch-sur-Alzette, Luxembourg; Wim Ammerlann, Luxembourg Institute of Health, Strassen, Luxembourg; Giuseppe Arena, Luxembourg Centre for Systems Biomedicine, University of Luxembourg, Esch-sur-Alzette, Luxembourg; Michele Bassis, Luxembourg Centre for Systems Biomedicine, University of Luxembourg, Esch-sur-Alzette, Luxembourg; Roxane Batutu, Centre Hospitalier de Luxembourg, Strassen, Luxembourg; Katy Beaumont, Luxembourg Institute of Health, Strassen, Luxembourg; Sibylle Béchet, Centre Hospitalier de Luxembourg, Strassen, Luxembourg; Guy Berchem, Centre Hospitalier de Luxembourg, Strassen, Luxembourg; Alexandre Bisdorff, Centre Hospitalier Emile Mayrisch, Esch-sur-Alzette, Luxembourg; Ibrahim Boussaad, Luxembourg Centre for Systems Biomedicine, University of Luxembourg, Esch-sur-Alzette, Luxembourg; David Bouvier, Laboratoire National de Santé, Dudelange, Luxembourg; Lorieza Castillo, Luxembourg Institute of Health, Strassen, Luxembourg; Gessica Contesotto, Luxembourg Institute of Health, Strassen, Luxembourg; Nancy De Bremaeker, Centre Hospitalier de Luxembourg, Strassen, Luxembourg; Brian Dewitt, Luxembourg Institute of Health, Strassen, Luxembourg; Nico Diederich, Centre Hospitalier de Luxembourg, Strassen, Luxembourg; Rene Dondelinger, Centre Hospitalier Emile Mayrisch, Esch-sur-Alzette, Luxembourg; Angelo Ferrari, Luxembourg Institute of Health, Strassen, Luxembourg; Katrin Frauenknecht, Laboratoire National de Santé, Dudelange, Luxembourg; Joëlle Fritz, Luxembourg Institute of Health, Strassen, Luxembourg; Carlos Gamio, Luxembourg Institute of Health, Strassen, Luxembourg; Manon Gantenbein, Luxembourg Institute of Health, Strassen, Luxembourg; Piotr Gawron, Luxembourg Centre for Systems Biomedicine, University of Luxembourg, Esch-sur-Alzette, Luxembourg; Laura Georges, Luxembourg Institute of Health, Strassen, Luxembourg; Soumyabrata Ghosh, Luxembourg Centre for Systems Biomedicine, University of Luxembourg, Esch-sur-Alzette, Luxembourg; Marijus Giraitis, Luxembourg Institute of Health, Strassen, Luxembourg, Centre Hospitalier de Luxembourg, Strassen, Luxembourg; Enrico Glaab, Luxembourg Centre for Systems Biomedicine, University of Luxembourg, Esch-sur-Alzette, Luxembourg; Martine Goergen, Centre Hospitalier de Luxembourg, Strassen, Luxembourg; Elisa Gómez de Lope, Luxembourg Centre for Systems Biomedicine, University of Luxembourg, Esch-sur-Alzette, Luxembourg; Jérôme Graas, Luxembourg Institute of Health, Strassen, Luxembourg; Mariella Graziano, Association of Physiotherapists in Parkinson's Disease Europe, Esch-sur-Alzette, Luxembourg; Valentin Groues, Luxembourg Centre for Systems Biomedicine, University of Luxembourg, Esch-sur-Alzette, Luxembourg; Anne Grünewald, Luxembourg Centre for Systems Biomedicine, University of Luxembourg, Esch-sur-Alzette, Luxembourg; Gaël Hammot, Luxembourg Institute of Health, Strassen, Luxembourg; Anne-Marie Hanff, Luxembourg Institute of Health, Strassen, Luxembourg, Faculty of Science, Technology and Medicine, University of Luxembourg, Esch-sur-Alzette, Luxembourg, Department of Epidemiology, CAPHRI School for Public Health and Primary Care, Maastricht University Medical Centre+, Maastricht, the Netherlands; Linda Hansen, Centre Hospitalier de Luxembourg, Strassen, Luxembourg; Estelle Henry, Luxembourg Institute of Health, Strassen, Luxembourg; Margaux Henry, Luxembourg Institute of Health, Strassen, Luxembourg; Sylvia Herbrink, Centre Hospitalier de Luxembourg, Strassen, Luxembourg; Sascha Herzinger, Luxembourg Centre for Systems Biomedicine, University of Luxembourg, Esch-sur-Alzette, Luxembourg; Alexander Hundt, Luxembourg Institute of Health, Strassen, Luxembourg; Jade Jaber, Luxembourg Centre for Systems Biomedicine, University of Luxembourg, Esch-sur-Alzette, Luxembourg, Centre Hospitalier de Luxembourg, Strassen, Luxembourg; Nadine Jacoby, Private practice, Ettelbruck, Luxembourg; Sonja Jónsdóttir, Luxembourg Institute of Health, Strassen, Luxembourg; Jochen Klucken, Luxembourg Centre for Systems Biomedicine, University of Luxembourg, Esch-sur-Alzette, Luxembourg, Centre Hospitalier de Luxembourg, Strassen, Luxembourg; Olga Kofanova, Luxembourg Institute of Health, Strassen, Luxembourg; Rejko Krüger, Luxembourg Centre for Systems Biomedicine, University of Luxembourg, Esch-sur-Alzette, Luxembourg, Luxembourg Institute of Health, Strassen, Luxembourg, Centre Hospitalier de Luxembourg, Strassen, Luxembourg; Pauline Lambert, Luxembourg Institute of Health, Strassen, Luxembourg; Zied Landoulsi, Luxembourg Institute of Health, Strassen, Luxembourg; Roseline Lentz, Parkinson Luxembourg Association, Leudelange, Luxembourg; Ana Festas Lopes, Luxembourg Institute of Health, Strassen, Luxembourg; Victoria Lorentz, Luxembourg Institute of Health, Strassen, Luxembourg; Guilherme Marques, Luxembourg Institute of Health, Strassen, Luxembourg; Tainá M. Marques, Luxembourg Institute of Health, Strassen, Luxembourg; Patricia Martins Conde, Luxembourg Centre for Systems Biomedicine, University of Luxembourg, Esch-sur-Alzette, Luxembourg; Patrick May, Luxembourg Centre for Systems Biomedicine, University of Luxembourg, Esch-sur-Alzette, Luxembourg; Deborah McIntyre, Luxembourg Institute of Health, Strassen, Luxembourg; Chouaib Mediouni, Luxembourg Institute of Health, Strassen, Luxembourg; Francoise Meisch, Luxembourg Centre for Systems Biomedicine, University of Luxembourg, Esch-sur-Alzette, Luxembourg; Alexia Mendibide, Luxembourg Institute of Health, Strassen, Luxembourg; Myriam Menster, Luxembourg Institute of Health, Strassen, Luxembourg; Maura Minelli, Luxembourg Institute of Health, Strassen, Luxembourg; Michel Mittelbronn, Luxembourg Centre for Systems Biomedicine, University of Luxembourg, Esch-sur-Alzette, Luxembourg, Luxembourg Institute of Health, Strassen, Luxembourg, Laboratoire National de Santé, Dudelange, Luxembourg, Faculty of Science, Technology and Medicine, University of Luxembourg, Esch-sur-Alzette, Luxembourg, Luxembourg Center of Neuropathology, Dudelange, Luxembourg, Department of Life Sciences and Medicine, University of Luxembourg, Esch-sur-Alzette, Luxembourg; Saïda Mtimet, Luxembourg Institute of Health, Strassen, Luxembourg; Maeva Munsch, Luxembourg Institute of Health, Strassen, Luxembourg; Romain Nati, Centre Hospitalier de Luxembourg, Strassen, Luxembourg; Ulf Nehrbass, Luxembourg Institute of Health, Strassen, Luxembourg; Sarah Nickels, Luxembourg Centre for Systems Biomedicine, University of Luxembourg, Esch-sur-Alzette, Luxembourg; Jean-Paul Nicolay, Private practice, Luxembourg, Luxembourg; Maria Fernanda Niño Uribe, Luxembourg Centre for Systems Biomedicine, University of Luxembourg, Esch-sur-Alzette, Luxembourg, Centre Hospitalier de Luxembourg, Strassen, Luxembourg; Fozia Noor, Luxembourg Institute of Health, Strassen, Luxembourg; Jochen Ohnmacht, Luxembourg Institute of Health, Strassen, Luxembourg; Clarissa P. C. Gomes, Luxembourg Centre for Systems Biomedicine, University of Luxembourg, Esch-sur-Alzette, Luxembourg; Sinthuja Pachchek, Luxembourg Centre for Systems Biomedicine, University of Luxembourg, Esch-sur-Alzette, Luxembourg; Claire Pauly, Luxembourg Institute of Health, Strassen, Luxembourg, Centre Hospitalier de Luxembourg, Strassen, Luxembourg; Laure Pauly, Luxembourg Institute of Health, Strassen, Luxembourg, Faculty of Science, Technology and Medicine, University of Luxembourg, Esch-sur-Alzette, Luxembourg; Lukas Pavelka, Luxembourg Institute of Health, Strassen, Luxembourg, Centre Hospitalier de Luxembourg, Strassen, Luxembourg; Magali Perquin, Luxembourg Institute of Health, Strassen, Luxembourg; Achilleas Pexaras, Luxembourg Institute of Health, Strassen, Luxembourg; Armin Rauschenberger, Luxembourg Centre for Systems Biomedicine, University of Luxembourg, Esch-sur-Alzette, Luxembourg, Luxembourg Institute of Health, Strassen, Luxembourg; Dheeraj Reddy Bobbili, Luxembourg Centre for Systems Biomedicine, University of Luxembourg, Esch-sur-Alzette, Luxembourg; Lucie Remark, Luxembourg Institute of Health, Strassen, Luxembourg; Ilsé Richard, Luxembourg Institute of Health, Strassen, Luxembourg; Olivia Roland, Luxembourg Institute of Health, Strassen, Luxembourg; Kirsten Roomp, Luxembourg Centre for Systems Biomedicine, University of Luxembourg, Esch-sur-Alzette, Luxembourg; Eduardo Rosales, Luxembourg Institute of Health, Strassen, Luxembourg; Stefano Sapienza, Luxembourg Centre for Systems Biomedicine, University of Luxembourg, Esch-sur-Alzette, Luxembourg; Venkata Satagopam, Luxembourg Centre for Systems Biomedicine, University of Luxembourg, Esch-sur-Alzette, Luxembourg; Sabine Schmitz, Luxembourg Centre for Systems Biomedicine, University of Luxembourg, Esch-sur-Alzette, Luxembourg; Reinhard Schneider, Luxembourg Centre for Systems Biomedicine, University of Luxembourg, Esch-sur-Alzette, Luxembourg; Jens Schwamborn, Luxembourg Centre for Systems Biomedicine, University of Luxembourg, Esch-sur-Alzette, Luxembourg; Raquel Severino, Luxembourg Institute of Health, Strassen, Luxembourg; Ruxandra Soare, Luxembourg Centre for Systems Biomedicine, University of Luxembourg, Esch-sur-Alzette, Luxembourg; Ekaterina Soboleva, Luxembourg Centre for Systems Biomedicine, University of Luxembourg, Esch-sur-Alzette, Luxembourg, Centre Hospitalier de Luxembourg, Strassen, Luxembourg; Kate Sokolowska, Luxembourg Institute of Health, Strassen, Luxembourg; Michael T. Heneka, Luxembourg Centre for Systems Biomedicine, University of Luxembourg, Esch-sur-Alzette, Luxembourg; Maud Theresine, Luxembourg Institute of Health, Strassen, Luxembourg; Hermann Thien, Luxembourg Institute of Health, Strassen, Luxembourg; Elodie Thiry, Centre Hospitalier de Luxembourg, Strassen, Luxembourg; Rebecca Ting Jiin Loo, Luxembourg Centre for Systems Biomedicine, University of Luxembourg, Esch-sur-Alzette, Luxembourg; Johanna Trouet, Luxembourg Institute of Health, Strassen, Luxembourg; Olena Tsurkalenko, Luxembourg Institute of Health, Strassen, Luxembourg; Michel Vaillant, Luxembourg Institute of Health, Strassen, Luxembourg; Carlos Vega, Luxembourg Institute of Health, Strassen, Luxembourg; Liliana Vilas Boas, Centre Hospitalier de Luxembourg, Strassen, Luxembourg; Paul Wilmes, Luxembourg Centre for Systems Biomedicine, University of Luxembourg, Esch-sur-Alzette, Luxembourg; Evi Wollscheid-Lengeling, Luxembourg Centre for Systems Biomedicine, University of Luxembourg, Esch-sur-Alzette, Luxembourg; Iñigo Yoldi Bergua, Luxembourg Centre for Systems Biomedicine, University of Luxembourg, Esch-sur-Alzette, Luxembourg; Gelani Zelimkhanov, Centre Hospitalier de Luxembourg, Strassen, Luxembourg.

## Author disclaimer

For the purpose of open access, and in fulfilment of the obligations arising from the grant agreement, the author has applied a Creative Commons Attribution 4.0 International (CC BY 4.0) license to any Author Accepted Manuscript version arising from this submission.
