# Supplementary material for: Progression of GBA1 severe and risk variants: a longitudinal mixed model analysis
Source: Front Aging Neurosci. 2026 Mar 26;18:1771789. doi: 10.3389/fnagi.2026.1771789 (PMC13062167; doi:10.3389/fnagi.2026.1771789)
Supplement: Supplementary file 1 [file Data_sheet_1.docx]

# Supplementary material

Progression of *GBA1* severe and risk variants: A longitudinal mixed model analysis

## 1. Supplementary tables

Table S1: Variants in the GBA1 gene detected in PD patients stratified by allele

| **Variant** | **Legacy name** | **Homo-zygote wildtype/reference** | **Hetero- zygote**  (n = 74) | **Homo- zygote**  (n = 2) |
| --- | --- | --- | --- | --- |
| **PD-risk** |  |  |  |  |
| p.T408M | T369M | 713 | 18 | 0 |
| p.E365K | E326K | 700 | 28 | ^a^2 |
| *Total* |  | 682 | 46 | 2 |
| **Severe** |  |  |  |  |
| p.G234W | G195W | 729 | 1 | 0 |
| p.G241R | G202R | 728 | 2 | 0 |
| p.G416S | G377S | 729 | 1 | 0 |
| p.L483P | L444P | 718 | 12 | 0 |
| p.P161S | P122S | 728 | 2 | 0 |
| p.R398* | R359X | 729 | 1 | 0 |
| p.R502H | R463H | 729 | 1 | 0 |
| p.F252I | F213I | 729 | 1 | 0 |
| c.115+1G>A | IVS2+1G>A | 729 | 1 | 0 |
| *Total* |  | 708 | 22 | 0 |

Note. ^a^As we found only 2 people with PD with homozygous p.E365K variants, we assumed a dominant model and combined individuals with heterozygous and homozygous variants into a variable.

Table S2: Variants in the GBA1 gene detected in PD patients stratified by PD severity (Hoglinger et al., 2022)

| **Markers** | **PD-risk** (n = 47) | **Severe**  (n = 22) |
| --- | --- | --- |
| p.E365K | 30 | 0 |
| p.T408M | 17^a^ | 0 |
| p.N409S | 0 | 0 |
| p.F252I | 0 | 1 |
| c.115+1G>A | 0 | 1 |
| p.G234W | 0 | 1 |
| p.G241R | 0 | 2 |
| p.G416S | 0 | 1 |
| p.L483P | 0 | 12 |
| p.P161S | 0 | 2 |
| p.R398X | 0 | 1 |
| p.R502H | 0 | 1 |

Note. ^a^ In one participant, the variant p.T408M (considered as PD-risk) were cooccured with p.F252I (severe). We allocated this participant to the group of people with a variant considered as severe as they are associated with a higher PD severity (Hoglinger et al., 2022).
^b^ In one participant variant p.N409S (considered as mild) were cooccuring p.L483P (severe). We allocated this participant to the group of people with a variant considered as severe as they are associated with a higher PD severity (Hoglinger et al., 2022).

Table S3: Characteristics of the study participants at baseline (N = 726) incl. numbers of missing data for each variable of interest

| **Characteristics** | **Mean (SD) / n (%)** | **Min. - Max.** | **Median (Pct25-75)** | **Missing N (%)** |
| --- | --- | --- | --- | --- |
| **Sociodemographic characteristics** | |  |  |  |
| Age (y.) | 67.2 (10.6) | 31.6 – 92.9 | 68.2 (60.2 – 74.7) | 0 (0%) |
| Male sex | 488 (66.6%) |  |  | 0 (0%) |
| Years of Education | 13.0 (4.1) | 1.0 – 30.0 | 13.0 (10.0 - 16.0) | 4 (0.5%) |
| Language most fluent |  |  |  | 0 (0%) |
| Luxembourgish | 316 (43.3%) |  |  |  |
| French | 204 (28.0%) |  |  |  |
| German | 118 (16.2%) |  |  |  |
| Other | 91 (12.5%) |  |  |  |
| Marital status |  |  |  | 3 (0.4%) |
| Single | 39 (5.3%) |  |  |  |
| Married / Partnered | 556 (75.9%) |  |  |  |
| Divorced / Bereaved | 135 (18.4%) |  |  |  |
| **Health-related characteristics** | |  |  |  |
| Diagnosis |  |  |  | 0 (0%) |
| Typical PD | 649 (88.5%) |  |  |  |
| PDD | 84 (11.5%) |  |  |  |
| Pathogenic *GBA1* carrier |  |  |  | 0 (0%) |
| No | 657 (89.6%) |  |  |  |
| Yes | 76 (10.4%) |  |  |  |
| Pathogenic *GBA1* variants |  |  |  | 0 (0%) |
| PD-Risk | 47 (61.8%) |  |  |  |
|  |  |  |  |  |
| Severe | 22 (28.9%) |  |  |  |
| Polygenic Risk Score | 0.2 (1.0) | -2.3 – 3.3 | 0.2 (-0.4 – 0.9) | 79 (10.8%) |
| Hoehn and Yahr (H&Y) Disease Stages |  |  |  | 16 (2.2%) |
| H&Y 1 | 76 (10.4%) |  |  |  |
| H&Y 1.5 | 60 (8.2%) |  |  |  |
| H&Y 2 | 362 (49.4%) |  |  |  |
| H&Y 2.5 | 100 (13.6%) |  |  |  |
| H&Y 3 | 69 (9.4%) |  |  |  |
| H&Y 4 | 36 (4.9%) |  |  |  |
| H&Y 5 | 14 (1.9%) |  |  |  |
| Disease duration (y.) | 4.9 (5.1) | 0.0 – 32.3 | 3.2 (1.1 – 7.4) | 48 (6.5%) |
| Age at diagnosis (y.) | 62.6 (11.5) | 30.0 – 91.0 | 63.0 (54.0 – 71.0) | 8 (1.1%) |
| Age at onset of motor-symptoms (y.) | 59.7 (12.5) | 17.0 – 88.0 | 61.0 (52.0 – 69.0) | 19 (2.6%) |
| Time to diagnosis (y.) | 2.7 (5.2) | -1.0 – 46.0 | 1.0 (0.0 – 3.0) | 25 (3.4%) |
| LEDD (mg.) | 491.3 (401.2) | 0.0 – 2062.0 | 400.0 (200.0 – 700.0) | 27 (3.7%) |
| PDQ-39 (0 – 100)^a^ | 24.5 (17.3) | 0.0 – 82.1 | 21.2 (10.9 – 34.0) | 59 (8.1%) |
| **Non-motor symptoms** |  |  |  |  |
| MoCA (0 – 30)^b^ | 24.6 (4.3) | 5.0 – 30.0 | 26.0 (23.0 – 28.0) | 18 (2.5%) |
| SAS (0 – 42)^a^ | 14.0 (5.9) | 1.0 – 16.0 | 13.0 (10.0 – 17.0) | 43 (5.9%) |
| BDI-I (0 – 63)^a^ | 9.8 (7.3) | 0.0 – 51.0 | 8.0 (4.8 – 14.0) | 37 (5.0%) |
| Sniffin’ sticks (0 - 16)^b^ | 8.1 (3.2) | 1.0 – 16.0 | 8.0 (6.0 – 10.0) | 49 (6.7%) |
| PDQ-39 subscale bodily discomfort (0 – 100)^a^ | 33.2 (23.9) | 0.0 – 100 | 33.3 (16.7 – 50.0) | 36 (4.9%) |
| PDSS (0 - 150)^b^ | 105.5 (24.8) | 17.0 – 150.0 | 108.5 (90.5 – 125.0) | 50 (6.8/%) |
| RBDSQ (0 - 13)^a^ | 4.6 (3.2) | 0.0 – 13.0 | 4.0 (2.0 – 7.0) | 52 (7.1%) |
| MDS-UPDRS I (0 – 52)^a^ | 10.4 (7.0) | 0.0 – 39.0 | 9.0 (5.0 - 14.0) | 25 (3.4%) |
| **Motor symptoms** |  |  |  |  |
| MDS-UPDRS II (0 – 52)^a^ | 11.1 (8.4) | 0.0 – 48.0 | 9.0 (5.0 - 15.0) | 18 (2.5%) |
| MDS-UPDRS III (0 – 132)^a^ | 34.0 (16.8) | 0.0 – 100.0 | 32.0 (22.0 - 44.0) | 20 (2.7%) |
| MDS-UPDRS IV (0 – 24)^a^ | 1.5 (3.2) | 0.0 – 16.0 | 0.0 (0.0 - 0.0) | 13 (1.8%) |
| FMCS (0 – 100)^b^ | 74.8 (23.0) | 1.6 – 100.0 | 81.2 (60.9 - 93.8) | 40 (5.5%) |
| PIGD Score (0 – 20)^a^ | 3.5 (3.7) | 0.0 – 20.0 | 2.0 (1.0 – 5.0) | 19 (2.6%) |
| Tremor Scale (0 - 4)^a^ | 0.6 (0.5) | 0.0 – 2.4 | 0.5 (0.3 – 0.8) | 16 (2.2%) |
| MDT Score (3 - 103)^a^ | 8.9 (9.2) | 0.0 – 56.0 | 6.0 (3.0 – 11.2) | 345 (47.1%) |

Note. SD: Standard Deviation, IQR: Interquartilerange, Pct: percentile, y: year, Time to diagnosis = Date of diagnosis – Date of first motor symptoms, *^a^ higher = worse, ^b^ higher = better.
Abbreviations: BDI-I: Beck Depression Inventory I, FMCS: Functional Mobility Composite Score, LEDD: Levodopa Equivalent Daily Dose, MDS: Movement Disorders Society, MDT: Munich Dysphagia Test, MoCA: Montreal Cognitive Assessment, PDQ39: Parkinson's Disease Questionnaire, PDSS: Parkinson's Disease Sleep Scale, PIGD: Postural Instabilities and Gait Disorders, RBDSQ: RBD Screening Questionnaire, SAS: Starkstein Apathy Scale, UPDRS: Unified Parkinson's Disease Rating Scale*

*Table S4: Fixed effects for the association of GBA1 variants with progression of non-motor symptoms*

|  | **SAS** | | | **MoCA** | | | **BDI-I** | | | **MDS-UPDRS I** | | |
| --- | --- | --- | --- | --- | --- | --- | --- | --- | --- | --- | --- | --- |
| *Predictors* | *stand. B (CI 95%)* | *P-Value* |  | *stand. B (CI 95%)* | *P-Value* |  | *stand. B (CI 95%)* | *P-Value* |  | *stand. B (CI 95%)* | *P-Value* |  |
| Intercept | -0.004  (-0.084 – 0.077) | 0.924 |  | 0.106  (0.018 – 0.194) | 0.018 |  | 0.065  (-0.008 – 0.138) | 0.080 |  | 0.085  (0.014 – 0.156) | 0.019 |  |
| Disease duration (y.) | 0.159  (0.090 – 0.227) | <0.001 |  | 0.286  (0.214 – 0.357) | <0.001 |  | 0.219  (0.151 – 0.288) | <0.001 |  | 0.366  (0.300 – 0.431) | <0.001 |  |
| Time to diagnosis (y.) | -0.021  (-0.093 – 0.052) | 0.577 |  | 0.005  (-0.066 – 0.077) | 0.890 |  | 0.014  (-0.055 – 0.084) | 0.689 |  | 0.013  (-0.056 – 0.082) | 0.706 |  |
| I(disease_duration^2) | 0.084  (0.046 – 0.123) | <0.001 |  | 0.082  (0.043 – 0.121) | <0.001 |  |  |  |  |  |  |  |
| **PD-Risk** |  |  |  |  |  |  |  |  |  |  |  |  |
| Disease duration: p.E365K  (n = 30) | 0.361  (0.020 – 0.702) | 0.038 |  | 0.316  (-0.052 – 0.685) | 0.092 |  | 0.229  (-0.130 – 0.588) | 0.210 |  | 0.371  (0.046 – 0.696) | 0.025 |  |
| Disease duration: p.T408M  (n = 17) | 0.411  (0.001 – 0.822) | 0.049 |  | 0.292  (-0.112 – 0.696) | 0.157 |  | -0.105  (-0.517 – 0.306) | 0.615 |  | 0.111  (-0.292 – 0.515) | 0.588 |  |
| **Severe** |  |  |  |  |  |  |  |  |  |  |  |  |
| Disease duration: p.F252I (n = 1) | -0.256  (-5.805 – 5.293) | 0.928 |  | -1.259  (-6.344 – 3.825) | 0.627 |  | 5.598  (-0.062 – 11.259) | 0.053 |  | -1.147  (-4.610 – 2.316) | 0.516 |  |
| Disease duration: p.G234W  (n = 1) | 0.935  (-0.448 – 2.318) | 0.185 |  | 1.555  (0.282 – 2.828) | 0.017 |  | 0.602  (-0.717 – 1.922) | 0.370 |  | 2.082  (0.790 – 3.375) | 0.002 |  |
| Disease duration: p.G241R  (n = 2) | -0.662  (-1.669 – 0.346) | 0.198 |  | 0.428  (-0.449 – 1.305) | 0.337 |  | -0.304  (-1.313 – 0.705) | 0.554 |  | -0.033  (-1.025 – 0.959) | 0.948 |  |
| Disease duration: p.G416S  (n = 1) | 0.908  (-1.046 – 2.861) | 0.362 |  | 3.030  (1.187 – 4.872) | 0.001 |  | 1.987  (-0.009 – 3.984) | 0.051 |  | 2.378  (0.378 – 4.378) | 0.020 |  |
| Disease duration: p.L483P (n = 12) | 0.599  (-0.040 – 1.237) | 0.066 |  | 0.353  (-0.373 – 1.079) | 0.340 |  | 0.772  (0.159 – 1.385) | 0.014 |  | -0.400  (-1.000 – 0.200) | 0.191 |  |
| Disease duration: p.P161S  (n = 2) | 2.431  (-2.152 – 7.014) | 0.298 |  | 1.701  (-2.762 – 6.164) | 0.455 |  | 0.956  (-3.556 – 5.468) | 0.678 |  | 1.277  (-2.105 – 4.658) | 0.459 |  |
| Disease duration: p.R398X  (n = 1) | -0.313  (-1.910 – 1.283) | 0.700 |  | -0.110  (-1.644 – 1.423) | 0.888 |  | -0.697  (-2.335 – 0.940) | 0.403 |  | 2.144  (0.532 – 3.756) | 0.009 |  |
| Disease duration: p.R502H  (n = 1) | 0.263  (-2.263 – 2.789) | 0.838 |  | 2.084  (-1.603 – 5.771) | 0.268 |  | 1.068  (-1.510 – 3.646) | 0.417 |  | 1.276  (-1.324 – 3.876) | 0.336 |  |
| **Random effects** |  |  |  |  |  |  |  |  |  |  |  |  |
| σ^2^ | 0.27 | | | 0.23 | | | 0.28 | | | 0.30 | | |
| τ_00_ | 0.68 _ND_ | | | 0.86 _ND_ | | | 0.58 _ND_ | | | 0.58 _ND_ | | |
| τ_11_ | 0.15 _ND.disease_duration_ | | | 0.18 _ND.disease_duration_ | | | 0.17 _ND.disease_duration_ | | | 0.14 _ND.disease_duration_ | | |
| ρ_01_ | 0.26 _ND_ | | | 0.67 _ND_ | | | 0.11 _ND_ | | | 0.21 _ND_ | | |
| ICC | 0.75 | | | 0.81 | | | 0.72 | | | 0.71 | | |
| N | 683 _ND_ | | | 687 _ND_ | | | 683 _ND_ | | | 694 _ND_ | | |
| Observations | 2562 | | | 2426 | | | 2456 | | | 2747 | | |
| Marginal R^2^ / Conditional R^2^ | 0.100 / 0.776 | | | 0.145 / 0.840 | | | 0.066 / 0.738 | | | 0.143 / 0.751 | | |
|  | | | | | | | | | | | | |

Note The main effects included in the model can be found on the OSF-project page: <https://osf.io/ncwta/>

*Table S4 (Continued)*

|  | **PDQ-39 Subscale bodily discomfort** | | | **Sniffin’ score** | | | **PDSS** | | | **RBDSQ** | | |
| --- | --- | --- | --- | --- | --- | --- | --- | --- | --- | --- | --- | --- |
| *Predictors* | *stand. B (CI 95%)* | *P-Value* |  | *stand. B (CI 95%)* | *P-Value* |  | *stand. B (CI 95%)* | *P-Value* |  | *stand. B (CI 95%)* | *P-Value* |  |
| Intercept | 0.112  (0.036 – 0.188) | 0.004 |  | 0.047  (-0.023 – 0.117) | 0.189 |  | 0.057  (-0.012 – 0.126) | 0.103 |  | 0.022  (-0.051 – 0.096) | 0.549 |  |
| Disease duration (y.) | 0.165  (0.102 – 0.228) | <0.001 |  | 0.196  (0.140 – 0.253) | <0.001 |  | 0.227  (0.170 – 0.285) | <0.001 |  | 0.189  (0.125 – 0.253) | <0.001 |  |
| Time to diagnosis (y.) | -0.016  (-0.084 – 0.053) | 0.656 |  | -0.027  (-0.097 – 0.042) | 0.438 |  | 0.022  (-0.045 – 0.090) | 0.515 |  | 0.077  (0.006 – 0.148) | 0.034 |  |
| I(disease_duration^2) | -0.058  (-0.093 – -0.023) | 0.001 |  |  |  |  |  |  |  |  |  |  |
| **PD-Risk** |  |  |  |  |  |  |  |  |  |  |  |  |
| Disease duration: p.E365K  (n = 30) | 0.033  (-0.269 – 0.335) | 0.830 |  | -0.266  (-0.536 – 0.004) | 0.054 |  | 0.371  (0.085 – 0.656) | 0.011 |  | -0.062  (-0.378 – 0.254) | 0.698 |  |
| Disease duration: p.T408M  (n = 17) | 0.027  (-0.350 – 0.404) | 0.889 |  | 0.175  (-0.179 – 0.529) | 0.332 |  | 0.019  (-0.344 – 0.383) | 0.917 |  | 0.089  (-0.294 – 0.472) | 0.649 |  |
| **Severe** |  |  |  |  |  |  |  |  |  |  |  |  |
| Disease duration: p.F252I (n = 1) | -1.452  (-7.585 – 4.681) | 0.643 |  | -0.196  (-6.091 – 5.698) | 0.948 |  | -0.149  (-6.296 – 5.998) | 0.962 |  |  |  |  |
| Disease duration: p.G234W  (n = 1) | 0.544  (-0.810 – 1.898) | 0.431 |  | -0.699  (-2.146 – 0.748) | 0.343 |  | 1.090  (-0.240 – 2.420) | 0.108 |  | 1.126  (-0.161 – 2.413) | 0.086 |  |
| Disease duration: p.G241R  (n = 2) | 0.780  (-0.167 – 1.726) | 0.106 |  | -0.907  (-1.832 – 0.018) | 0.055 |  | 0.255  (-0.664 – 1.175) | 0.586 |  | 0.071  (-0.871 – 1.013) | 0.883 |  |
| Disease duration: p.G416S  (n = 1) | 1.619  (-0.431 – 3.669) | 0.121 |  | -1.075  (-3.838 – 1.687) | 0.445 |  | 0.144  (-1.896 – 2.185) | 0.890 |  | -0.929  (-2.746 – 0.887) | 0.316 |  |
| Disease duration: p.L483P (n = 12) | -0.178  (-0.746 – 0.390) | 0.538 |  | -0.159  (-0.785 – 0.467) | 0.618 |  | -0.736  (-1.355 – -0.118) | 0.020 |  | -0.366  (-0.966 – 0.235) | 0.232 |  |
| Disease duration: p.P161S  (n = 2) | 1.272  (-3.457 – 6.001) | 0.598 |  | -0.614  (-5.157 – 3.929) | 0.791 |  | 2.339  (-2.319 – 6.998) | 0.325 |  | 2.657  (-1.628 – 6.942) | 0.224 |  |
| Disease duration: p.R398X  (n = 1) | 1.699  (0.082 – 3.316) | 0.039 |  | 1.157  (-0.467 – 2.780) | 0.163 |  | -0.598  (-2.193 – 0.996) | 0.462 |  | -1.573  (-3.052 – -0.095) | 0.037 |  |
| Disease duration: p.R502H  (n = 1) | -0.993  (-3.710 – 1.723) | 0.473 |  | -1.088  (-5.310 – 3.133) | 0.613 |  | -0.165  (-2.877 – 2.547) | 0.905 |  | -0.440  (-2.783 – 1.904) | 0.713 |  |
| **Random effects** |  |  |  |  |  |  |  |  |  |  |  |  |
| σ^2^ | 0.34 | | | 0.31 | | | 0.34 | | | 0.23 | | |
| τ_00_ | 0.59 _ND_ | | | 0.57 _ND_ | | | 0.55 _ND_ | | | 0.63 _ND_ | | |
| τ_11_ | 0.06 _ND.disease_duration_ | | | 0.03 _ND.disease_duration_ | | | 0.05 _ND.disease_duration_ | | | 0.14 _ND.disease_duration_ | | |
| ρ_01_ | -0.04 _ND_ | | | -0.43 _ND_ | | | -0.25 _ND_ | | | 0.13 _ND_ | | |
| ICC | 0.66 | | | 0.66 | | | 0.64 | | | 0.77 | | |
| N | 689 _ND_ | | | 666 _ND_ | | | 685 _ND_ | | | 681 _ND_ | | |
| Observations | 2603 | | | 2243 | | | 2554 | | | 2530 | | |
| Marginal R^2^ / Conditional R^2^ | 0.041 / 0.670 | | | 0.055 / 0.678 | | | 0.069 / 0.666 | | | 0.055 / 0.778 | | |
|  | | | | | | | | | | | | |

Note The main effects included in the model can be found on the OSF-project page: <https://osf.io/ncwta/>

*Table S5: Fixed effects for the association of GBA1 variants with progression of motor symptoms*

|  | **FMCS** | | | **MDS-UPDRS II** | | | **MDS-UPDRS III** | | | **MDS-UPDRS IV** | | |
| --- | --- | --- | --- | --- | --- | --- | --- | --- | --- | --- | --- | --- |
| *Predictors* | *stand. B (CI 95%)* | *P-Value* |  | *stand. B (CI 95%)* | *P-Value* |  | *stand. B (CI 95%)* | *P-Value* |  | *stand. B (CI 95%)* | *P-Value* |  |
| Intercept | 0.177  (0.095 – 0.259) | <0.001 |  | 0.164  (0.085 – 0.244) | <0.001 |  | 0.140  (0.062 – 0.219) | <0.001 |  | 0.093  (0.023 – 0.162) | 0.009 |  |
| Disease duration (y.) | 0.555  (0.480 – 0.630) | <0.001 |  | 0.687  (0.617 – 0.757) | <0.001 |  | 0.395  (0.325 – 0.466) | <0.001 |  | 0.400  (0.332 – 0.469) | <0.001 |  |
| I(disease_duration^2) |  |  |  |  |  |  | -0.014  (-0.055 – 0.028) | 0.523 |  | -0.087  (-0.129 – -0.045) | <0.001 |  |
| Time to diagnosis (y.) | 0.019  (-0.051 – 0.088) | 0.599 |  | 0.017  (-0.048 – 0.082) | 0.607 |  | 0.049  (-0.017 – 0.114) | 0.144 |  | 0.003  (-0.041 – 0.048) | 0.880 |  |
| Disease duration: p.E365K (n = 30) | 0.143  (-0.231 – 0.517) | 0.453 |  | 0.075  (-0.277 – 0.427) | 0.675 |  | 0.160  (-0.182 – 0.502) | 0.359 |  | -0.104  (-0.448 – 0.240) | 0.553 |  |
| Disease duration: p.T408M (n = 17) | 0.036  (-0.405 – 0.477) | 0.874 |  | 0.106  (-0.316 – 0.528) | 0.621 |  | -0.039  (-0.455 – 0.377) | 0.854 |  | -0.280  (-0.693 – 0.133) | 0.183 |  |
| Disease duration: p.N409S (n = 7) | -0.061  (-0.764 – 0.642) | 0.865 |  | -0.359  (-1.018 – 0.299) | 0.285 |  | -0.194  (-0.871 – 0.482) | 0.573 |  | -0.366  (-1.081 – 0.349) | 0.315 |  |
| Disease duration: p.F252I (n = 1) | 1.004  (-3.325 – 5.333) | 0.649 |  | -0.483  (-3.347 – 2.382) | 0.741 |  |  |  |  | 1.002  (-3.259 – 5.263) | 0.645 |  |
| Disease duration: p.G234W (n = 1) | -0.089  (-1.560 – 1.382) | 0.905 |  | 0.232  (-1.119 – 1.583) | 0.736 |  | 0.239  (-1.125 – 1.604) | 0.730 |  | -1.843  (-3.373 – -0.313) | 0.018 |  |
| Disease duration: p.G241R (n = 2) | -0.019  (-1.096 – 1.058) | 0.972 |  | -0.351  (-1.383 – 0.681) | 0.505 |  | 0.461  (-0.445 – 1.367) | 0.317 |  | 0.282  (-0.655 – 1.220) | 0.554 |  |
| Disease duration: p.G416S (n = 1) | 1.365  (-0.512 – 3.241) | 0.154 |  | 1.506  (-0.300 – 3.312) | 0.102 |  | 0.348  (-1.788 – 2.485) | 0.749 |  | 0.242  (-2.186 – 2.669) | 0.845 |  |
| Disease duration: p.L483P (n = 12) | 0.094  (-0.545 – 0.733) | 0.772 |  | 0.458  (-0.123 – 1.038) | 0.122 |  | 0.532  (-0.186 – 1.250) | 0.147 |  | 0.361  (-0.238 – 0.960) | 0.238 |  |
| Disease duration: p.P161S (n = 2) | 2.432  (-1.451 – 6.315) | 0.220 |  | 3.744  (0.612 – 6.876) | 0.019 |  | 4.896  (0.057 – 9.735) | 0.047 |  | 0.645  (-3.168 – 4.459) | 0.740 |  |
| Disease duration: p.R398X (n = 1) | 1.887  (0.301 – 3.474) | 0.020 |  | 1.735  (0.187 – 3.283) | 0.028 |  | 0.633  (-1.089 – 2.355) | 0.471 |  | 3.170  (1.225 – 5.116) | 0.001 |  |
| Disease duration: p.R502H (n = 1) | 2.052  (-0.122 – 4.227) | 0.064 |  | -0.100  (-2.329 – 2.129) | 0.930 |  | 0.923  (-1.868 – 3.715) | 0.517 |  | -0.573  (-5.687 – 4.540) | 0.826 |  |
| **Random effects** |  |  |  |  |  |  |  |  |  |  |  |  |
| σ^2^ | 0.16 | | | 0.18 | | | 0.35 | | | 0.45 | | |
| τ_00_ | 0.79 _ND_ | | | 0.77 _ND_ | | | 0.57 _ND_ | | | 0.37 _ND_ | | |
| τ_11_ | 0.36 _ND.disease_duration_ | | | 0.28 _ND.disease_duration_ | | | 0.13 _ND.disease_duration_ | | | 0.17 _ND.disease_duration_ | | |
| ρ_01_ | 0.56 _ND_ | | | 0.67 _ND_ | | | 0.49 _ND_ | | | 1.00 _ND_ | | |
| ICC | 0.88 | | | 0.85 | | | 0.66 | | |  | | |
| N | 685 _ND_ | | | 696 _ND_ | | | 693 _ND_ | | | 698 _ND_ | | |
| Observations | 2578 | | | 2788 | | | 2459 | | | 2810 | | |
| Marginal R^2^ / Conditional R^2^ | 0.196 / 0.901 | | | 0.280 / 0.893 | | | 0.133 / 0.709 | | | 0.206 / NA | | |
|  | | | | | | | | | | | | |

Note The main effects included in the model can be found on the OSF-project page: <https://osf.io/ncwta/>

*Table S5 (Continued)*

|  | **PIGD** | | | **Tremore scale** | | | **MDT** | | |
| --- | --- | --- | --- | --- | --- | --- | --- | --- | --- |
| *Predictors* | *stand. B (CI 95%)* | *P-Value* |  | *stand. B (CI 95%)* | *P-Value* |  | *stand. B (CI 95%)* | *P-Value* |  |
| Intercept | 0.123  (0.036 – 0.211) | 0.006 |  | 0.012  (-0.063 – 0.087) | 0.760 |  | 0.048  (-0.032 – 0.128) | 0.239 |  |
| Disease duration (y.) | 0.672  (0.593 – 0.751) | <0.001 |  | -0.160  (-0.228 – -0.092) | <0.001 |  | 0.356  (0.280 – 0.432) | <0.001 |  |
| I(disease_duration^2) | 0.158  (0.118 – 0.198) | <0.001 |  |  |  |  |  |  |  |
| Time to diagnosis (y.) | 0.007  (-0.055 – 0.069) | 0.820 |  | 0.088  (0.016 – 0.159) | 0.017 |  | 0.055  (-0.017 – 0.127) | 0.132 |  |
| Disease duration: p.E365K (n = 30) | -0.155  (-0.551 – 0.241) | 0.442 |  | 0.305  (-0.022 – 0.632) | 0.067 |  | 0.039  (-0.354 – 0.433) | 0.845 |  |
| Disease duration: p.T408M (n = 17) | -0.051  (-0.512 – 0.410) | 0.829 |  | 0.150  (-0.259 – 0.558) | 0.472 |  | 0.057  (-0.375 – 0.488) | 0.796 |  |
| Disease duration: p.N409S (n = 7) | -0.381  (-1.111 – 0.348) | 0.305 |  | -0.221  (-0.858 – 0.416) | 0.496 |  | -0.261  (-0.975 – 0.453) | 0.473 |  |
| Disease duration: p.F252I (n = 1) |  |  |  | 3.319  (-2.889 – 9.527) | 0.295 |  |  |  |  |
| Disease duration: p.G234W (n = 1) | -0.944  (-2.436 – 0.548) | 0.214 |  | -0.400  (-1.703 – 0.904) | 0.547 |  | 2.644  (0.466 – 4.822) | 0.017 |  |
| Disease duration: p.G241R (n = 2) | -0.623  (-1.748 – 0.501) | 0.276 |  | 0.822  (-0.181 – 1.825) | 0.108 |  | 0.114  (-0.880 – 1.108) | 0.822 |  |
| Disease duration: p.G416S (n = 1) | 0.347  (-1.629 – 2.323) | 0.730 |  | -1.558  (-3.663 – 0.547) | 0.147 |  | 0.483  (-1.402 – 2.368) | 0.615 |  |
| Disease duration: p.L483P (n = 12) | 0.028  (-0.712 – 0.768) | 0.941 |  | -0.189  (-0.862 – 0.485) | 0.582 |  | 0.274  (-0.439 – 0.987) | 0.451 |  |
| Disease duration: p.P161S (n = 2) | 9.391  (4.999 – 13.783) | <0.001 |  | -0.148  (-5.009 – 4.714) | 0.952 |  | 2.743  (-1.681 – 7.167) | 0.224 |  |
| Disease duration: p.R398X (n = 1) | 0.317  (-1.392 – 2.027) | 0.715 |  | -0.377  (-2.046 – 1.292) | 0.658 |  | 1.696  (-0.280 – 3.673) | 0.092 |  |
| Disease duration: p.R502H (n = 1) | 1.783  (-0.651 – 4.217) | 0.151 |  | 0.093  (-2.676 – 2.862) | 0.948 |  | 1.355  (-1.059 – 3.769) | 0.271 |  |
| **Continued** |  |  |  |  |  |  |  |  |  |
| σ^2^ | 0.22 | | | 0.35 | | | 0.24 | | |
| τ_00_ | 0.83 _ND_ | | | 0.64 _ND_ | | | 0.68 _ND_ | | |
| τ_11_ | 0.34 _ND.disease_duration_ | | | 0.10 _ND.disease_duration_ | | | 0.17 _ND.disease_duration_ | | |
| ρ_01_ | 0.79 _ND_ | | | 0.12 _ND_ | | | 0.47 _ND_ | | |
| ICC | 0.84 | | | 0.68 | | | 0.77 | | |
| N | 693 _ND_ | | | 695 _ND_ | | | 630 _ND_ | | |
| Observations | 2430 | | | 2435 | | | 2055 | | |
| Marginal R^2^ / Conditional R^2^ | 0.371 / 0.897 | | | 0.040 / 0.692 | | | 0.123 / 0.803 | | |
|  | | | | | | | | | |

Note The main effects included in the model can be found on the OSF-project page: <https://osf.io/ncwta/>

*Table S6: Fixed effects for the association of GBA1 variants considered as risk or severe with progression of non-motor symptoms*

|  | **SAS** | | | **MoCA** | | | **BDI-I** | | | **MDS-UPDRS I** | | |
| --- | --- | --- | --- | --- | --- | --- | --- | --- | --- | --- | --- | --- |
| *Predictors* | *stand. B (CI 95%)* | *P-Value* |  | *stand. B (CI 95%)* | *P-Value* |  | *stand. B (CI 95%)* | *P-Value* |  | *stand. B (CI 95%)* | *P-Value* |  |
| Intercept | -0.004  (-0.084 – 0.076) | 0.922 |  | 0.105  (0.017 – 0.193) | 0.019 |  | 0.066  (-0.008 – 0.139) | 0.080 |  | 0.086  (0.014 – 0.157) | 0.019 |  |
| Disease duration (y.) | 0.160  (0.092 – 0.229) | <0.001 |  | 0.288  (0.217 – 0.359) | <0.001 |  | 0.222  (0.153 – 0.291) | <0.001 |  | 0.366  (0.301 – 0.431) | <0.001 |  |
| Time to diagnosis (y.) | -0.021  (-0.094 – 0.051) | 0.564 |  | 0.005  (-0.067 – 0.076) | 0.894 |  | 0.015  (-0.055 – 0.084) | 0.680 |  | 0.015  (-0.054 – 0.084) | 0.662 |  |
| I(disease_duration^2) | 0.086  (0.048 – 0.124) | <0.001 |  | 0.086  (0.048 – 0.124) | <0.001 |  |  |  |  |  |  |  |
| Disease duration: PD-risk variants | 0.380  (0.115 – 0.645) | 0.005 |  | 0.291  (0.014 – 0.567) | 0.039 |  | 0.076  (-0.200 – 0.352) | 0.589 |  | 0.270  (0.014 – 0.526) | 0.039 |  |
| Disease duration: Severe variants | 0.239  (-0.167 – 0.645) | 0.248 |  | 0.614  (0.193 – 1.036) | 0.004 |  | 0.368  (-0.031 – 0.768) | 0.071 |  | 0.121  (-0.264 – 0.506) | 0.537 |  |
| **Random effects** |  |  |  |  |  |  |  |  |  |  |  |  |
| σ^2^ | 0.27 | | | 0.23 | | | 0.29 | | | 0.30 | | |
| τ_00_ | 0.69 _ND_ | | | 0.89 _ND_ | | | 0.60 _ND_ | | | 0.59 _ND_ | | |
| τ_11_ | 0.16 _ND.disease_duration_ | | | 0.19 _ND.disease_duration_ | | | 0.18 _ND.disease_duration_ | | | 0.15 _ND.disease_duration_ | | |
| ρ_01_ | 0.27 _ND_ | | | 0.67 _ND_ | | | 0.16 _ND_ | | | 0.22 _ND_ | | |
| ICC | 0.75 | | | 0.82 | | | 0.73 | | | 0.71 | | |
| N | 683 _ND_ | | | 687 _ND_ | | | 683 _ND_ | | | 694 _ND_ | | |
| Observations | 2562 | | | 2426 | | | 2456 | | | 2747 | | |
| Marginal R^2^ / Conditional R^2^ | 0.094 / 0.777 | | | 0.142 / 0.842 | | | 0.062 / 0.744 | | | 0.135 / 0.750 | | |
|  | | | | | | | | | | | | |

*Table S6 (Continued)*

|  | **PDQ-39 Subscale bodily discomfort** | | | **Sniffin’ sticks** | | | **PDSS** | | | **RBDSQ** | | |
| --- | --- | --- | --- | --- | --- | --- | --- | --- | --- | --- | --- | --- |
| *Predictors* | *stand. B (CI 95%)* | *P-Value* |  | *stand. B (CI 95%)* | *P-Value* |  | *stand. B (CI 95%)* | *P-Value* |  | *stand. B (CI 95%)* | *P-Value* |  |
| Intercept | 0.111  (0.035 – 0.187) | 0.004 |  | 0.047  (-0.023 – 0.117) | 0.187 |  | 0.058  (-0.011 – 0.126) | 0.100 |  | 0.022  (-0.051 – 0.096) | 0.549 |  |
| Disease duration (y.) | 0.165  (0.101 – 0.228) | <0.001 |  | 0.197  (0.141 – 0.254) | <0.001 |  | 0.227  (0.170 – 0.285) | <0.001 |  | 0.189  (0.125 – 0.253) | <0.001 |  |
| Time to diagnosis (y.) | -0.015  (-0.084 – 0.055) | 0.678 |  | -0.027  (-0.097 – 0.043) | 0.452 |  | 0.024  (-0.044 – 0.091) | 0.492 |  | 0.076  (0.005 – 0.146) | 0.036 |  |
| I(disease_duration^2) | -0.056  (-0.091 – -0.021) | 0.002 |  |  |  |  |  |  |  |  |  |  |
| Disease duration: PD-risk variants | 0.051  (-0.190 – 0.291) | 0.680 |  | -0.107  (-0.326 – 0.111) | 0.334 |  | 0.244  (0.017 – 0.471) | 0.035 |  | 0.002  (-0.244 – 0.248) | 0.988 |  |
| Disease duration: Severe variants | 0.157  (-0.202 – 0.517) | 0.391 |  | -0.119  (-0.460 – 0.223) | 0.495 |  | -0.176  (-0.523 – 0.170) | 0.317 |  | -0.073  (-0.455 – 0.309) | 0.706 |  |
| **Random effects** |  |  |  |  |  |  |  |  |  |  |  |  |
| σ^2^ | 0.34 | | | 0.31 | | | 0.34 | | | 0.24 | | |
| τ_00_ | 0.60 _ND_ | | | 0.58 _ND_ | | | 0.56 _ND_ | | | 0.64 _ND_ | | |
| τ_11_ | 0.07 _ND.disease_duration_ | | | 0.03 _ND.disease_duration_ | | | 0.06 _ND.disease_duration_ | | | 0.14 _ND.disease_duration_ | | |
| ρ_01_ | 0.00 _ND_ | | | -0.41 _ND_ | | | -0.25 _ND_ | | | 0.13 _ND_ | | |
| ICC | 0.66 | | | 0.66 | | | 0.64 | | | 0.77 | | |
| N | 689 _ND_ | | | 666 _ND_ | | | 685 _ND_ | | | 681 _ND_ | | |
| Observations | 2603 | | | 2243 | | | 2554 | | | 2530 | | |
| Marginal R^2^ / Conditional R^2^ | 0.023 / 0.672 | | | 0.044 / 0.679 | | | 0.060 / 0.664 | | | 0.043 / 0.777 | | |
|  | | | | | | | | | | | | |

Note The main effects included in the model can be found on the OSF-project page: <https://osf.io/ncwta/>

*Table S7: Fixed effects for the association of GBA1 variants considered as risk or severe with progression of motor symptoms*

|  | **FMCS** | | | **MDS-UPDRS II** | | | **MDS-UPDRS III** | | | **MDS-UPDRS IV** | | |
| --- | --- | --- | --- | --- | --- | --- | --- | --- | --- | --- | --- | --- |
| *Predictors* | *stand. B (CI 95%)* | *P-Value* |  | *stand. B (CI 95%)* | *P-Value* |  | *stand. B (CI 95%)* | *P-Value* |  | *stand. B (CI 95%)* | *P-Value* |  |
| Intercept | 0.177  (0.095 – 0.259) | <0.001 |  | 0.165  (0.085 – 0.244) | <0.001 |  | 0.140  (0.061 – 0.219) | 0.001 |  | 0.093  (0.024 – 0.162) | 0.009 |  |
| Disease duration (y.) | 0.555  (0.481 – 0.630) | <0.001 |  | 0.687  (0.618 – 0.757) | <0.001 |  | 0.396  (0.326 – 0.467) | <0.001 |  | 0.400  (0.332 – 0.469) | <0.001 |  |
| I(disease_duration^2) |  |  |  |  |  |  | -0.012  (-0.054 – 0.029) | 0.550 |  | -0.087  (-0.129 – -0.046) | <0.001 |  |
| Time to diagnosis (y.) | 0.020  (-0.049 – 0.089) | 0.573 |  | 0.018  (-0.047 – 0.082) | 0.594 |  | 0.049  (-0.016 – 0.115) | 0.138 |  | 0.004  (-0.040 – 0.049) | 0.847 |  |
| Disease duration: PD-risk variants | 0.109  (-0.178 – 0.397) | 0.455 |  | 0.090  (-0.183 – 0.362) | 0.518 |  | 0.081  (-0.186 – 0.348) | 0.552 |  | -0.174  (-0.442 – 0.093) | 0.201 |  |
| Disease duration: Severe variants | 0.216  (-0.208 – 0.640) | 0.317 |  | 0.284  (-0.108 – 0.676) | 0.156 |  | 0.218  (-0.193 – 0.629) | 0.298 |  | 0.246  (-0.131 – 0.624) | 0.200 |  |
| **Random effects** |  |  |  |  |  |  |  |  |  |  |  |  |
| σ^2^ | 0.16 | | | 0.18 | | | 0.35 | | | 0.46 | | |
| τ_00_ | 0.81 _ND_ | | | 0.78 _ND_ | | | 0.59 _ND_ | | | 0.37 _ND_ | | |
| τ_11_ | 0.36 _ND.disease_duration_ | | | 0.28 _ND.disease_duration_ | | | 0.14 _ND.disease_duration_ | | | 0.17 _ND.disease_duration_ | | |
| ρ_01_ | 0.56 _ND_ | | | 0.67 _ND_ | | | 0.51 _ND_ | | | 1.00 _ND_ | | |
| ICC | 0.88 | | | 0.85 | | | 0.67 | | |  | | |
| N | 685 _ND_ | | | 696 _ND_ | | | 693 _ND_ | | | 698 _ND_ | | |
| Observations | 2578 | | | 2788 | | | 2459 | | | 2810 | | |
| Marginal R^2^ / Conditional R^2^ | 0.198 / 0.902 | | | 0.284 / 0.893 | | | 0.133 / 0.714 | | | 0.191 / NA | | |
|  | | | | | | | | | | | | |

Note The main effects included in the model can be found on the OSF-project page: <https://osf.io/ncwta/>

*Table S7 (Continued)*

|  | **PIGD** | | | **Tremor scale** | | | **MDT** | | |
| --- | --- | --- | --- | --- | --- | --- | --- | --- | --- |
| *Predictors* | *stand. B (CI 95%)* | *P-Value* |  | *stand. B (CI 95%)* | *P-Value* |  | *stand. B (CI 95%)* | *P-Value* |  |
| Intercept | 0.124  (0.036 – 0.211) | 0.006 |  | 0.012  (-0.063 – 0.086) | 0.759 |  | 0.048  (-0.031 – 0.128) | 0.235 |  |
| Disease duration (y.) | 0.671  (0.593 – 0.749) | <0.001 |  | -0.160  (-0.228 – -0.093) | <0.001 |  | 0.356  (0.281 – 0.432) | <0.001 |  |
| I(disease_duration^2) | 0.157  (0.117 – 0.196) | <0.001 |  |  |  |  |  |  |  |
| Time to diagnosis (y.) | 0.007  (-0.055 – 0.069) | 0.821 |  | 0.089  (0.017 – 0.160) | 0.015 |  | 0.055  (-0.016 – 0.127) | 0.130 |  |
| Disease duration: PD-risk variants | -0.106  (-0.407 – 0.196) | 0.491 |  | 0.258  (0.001 – 0.515) | 0.050 |  | 0.047  (-0.245 – 0.340) | 0.750 |  |
| Disease duration: Severe variants | -0.122  (-0.576 – 0.332) | 0.598 |  | -0.077  (-0.474 – 0.320) | 0.702 |  | 0.363  (-0.068 – 0.794) | 0.098 |  |
| **Random effects** |  |  |  |  |  |  |  |  |  |
| σ^2^ | 0.22 | | | 0.35 | | | 0.24 | | |
| τ_00_ | 0.83 _ND_ | | | 0.64 _ND_ | | | 0.68 _ND_ | | |
| τ_11_ | 0.34 _ND.disease_duration_ | | | 0.11 _ND.disease_duration_ | | | 0.18 _ND.disease_duration_ | | |
| ρ_01_ | 0.79 _ND_ | | | 0.12 _ND_ | | | 0.47 _ND_ | | |
| ICC | 0.83 | | | 0.68 | | | 0.78 | | |
| N | 693 _ND_ | | | 695 _ND_ | | | 630 _ND_ | | |
| Observations | 2430 | | | 2435 | | | 2055 | | |
| Marginal R^2^ / Conditional R^2^ | 0.372 / 0.896 | | | 0.033 / 0.692 | | | 0.121 / 0.803 | | |
|  | | | | | | | | | |

Note The main effects included in the model can be found on the OSF-project page: <https://osf.io/ncwta/>

## Supplemental figures

Participants excluded with atypical PD:

n = 161 / 990 (16.3%)

Atypical PD: n = 159 / 990 (16.1%)

Others: n = 2 / 990 (0.002%)

Participants with PD and PDD

n = 829

**Included**

**Screening**

**Participants included in the analysis**n = 726

|  | Non-carriers | Risk | Severe |
| --- | --- | --- | --- |
| Baseline | 657 | 47 | 22 |
| Follow-up 1 | 540 | 42 | 19 |
| Follow-up 2 | 459 | 37 | 13 |
| Follow-up 3 | 361 | 28 | 12 |
| Follow-up 4 | 295 | 18 | 11 |
| Follow-up 5 | 192 | 12 | 6 |
| Follow-up 6 | 122 | 10 | 3 |
| Follow-up 7 | 77 | 7 | 2 |

All people with Parkinsonism at the NCER-PD cohort on 29/01/2024

n = 990

Participants excluded:

n = 96 / 829 (11.6%):

without consent for genetical analyses:
n = 6 / 829 (0.7%)

without PacBio: n = 57 / 829 (6.9%)

VUS: n = 21 / 829 (2.5%)

with other pathogenic variants:

CNV PRKN: n = 4 / 829 (0.5%)
LRRK2: n = 7 / 829 (0.8%)
PINK1: n = 1 / 829 (1.2%)

Figure S1: Flow diagram of patient recruitment


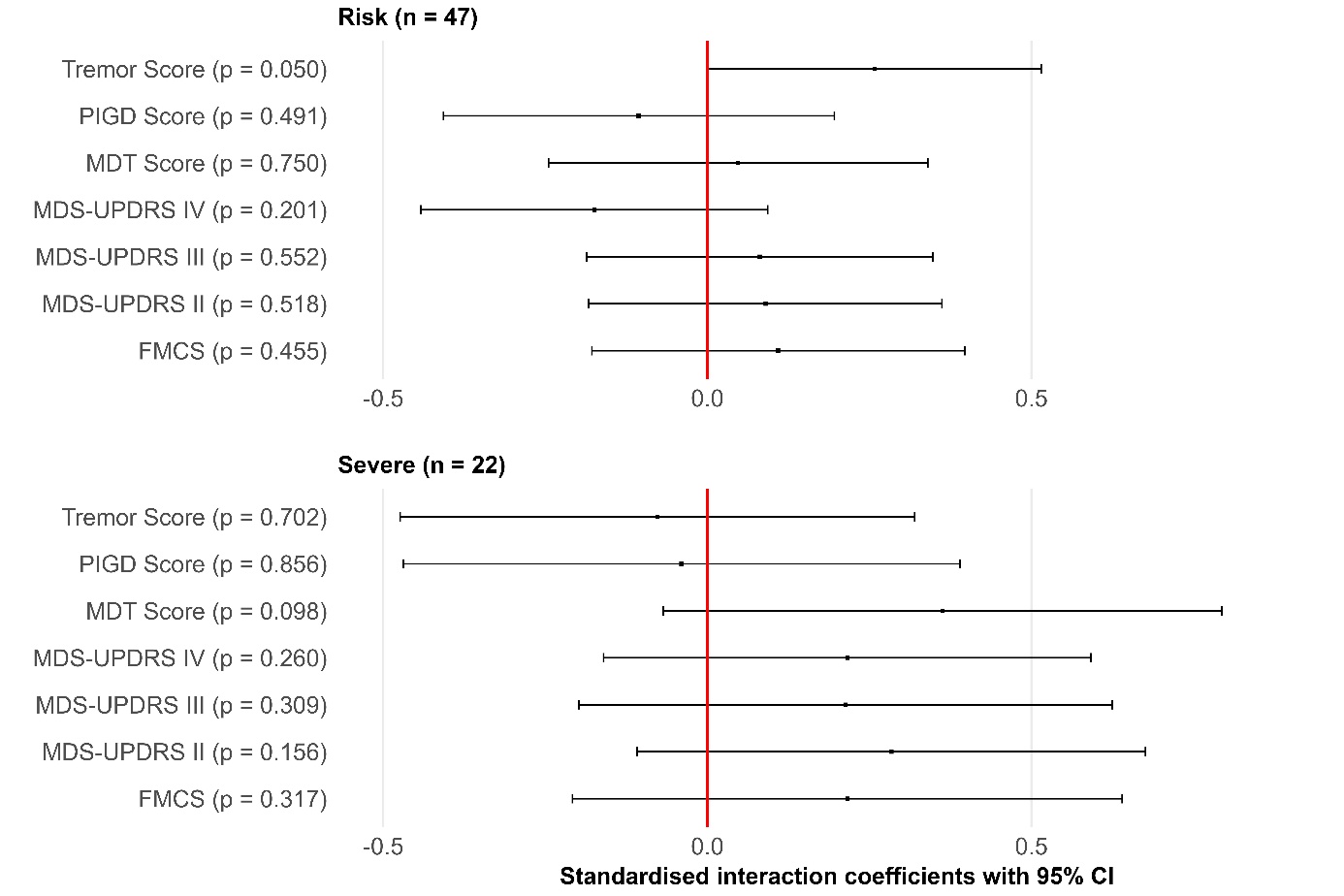


Figure S2: Association ofrisk and severe variants with progression of motor symptoms, right side of the red line = associated with worse progression compared to non-carriers. Abbreviations: FMCS: Functional Mobility Composite Score, MDS: Movement Disorders Society, MDT: Munich Dysphagia Test, PIGD: Postural Instabilities and Gait Disorders, RBDSQ: RBD Screening Questionnaire, UPDRS: Unified Parkinson's Disease Rating Scale

## 3. Description of the association of the different *GBA1* variants with progression of motor and non-motor symptoms

To analyse if different amino-acid changes are associated with a different effect of the time since diagnosis on motor- and non-motor symptoms we created one interaction model per outcome and added a categorical variable of twelve variants changes (see Table S2, reference group = non-carriers) as an interaction effect with the time since diagnosis and the outcome.

We reported the standardised interaction coefficients of the different variants in Tables S4 – S5 in the Supplement. Thus, no significant associations were detected after Bonferroni-adjusted significance level (alpha = 0.05/(15 outcomes12 variants) = 0.0003). On an unadjusted significance level (alpha = 0.05), while few in numbers, compared to non-carriers, the thirty carriers of at least one allele of p.E365K, a variant considered as risk-variant, were associated with a faster progression in apathy (SAS) (0.361, 95%CI: 0.020, 0.702, p = 0.038), non-motor symptoms (MDS-UPDRS I) (0.371, 95%CI: 0.046, 0.696, p = 0.025), and in quality of sleep (PDSS) (0.371, 95%CI: 0.085, 0.656, p = 0.011), while, compared to non-carriers, the seventeen carriers of p.T408M, a variant considered as PD-risk variant, were associated with a faster progression in apathy (SAS) only (0.411, 95%CI: 0.001, 0.822, p = 0.049). Finally, compared to non-carriers, the twelve carriers of at least one allele in variant p.L483P, a variant considered as severe variant, were associated with a faster progression in depression (BDI-I) (0.772, 95%CI: 0.159, 1.385, p = 0.014) and a slower worsening of quality of sleep (PDSS) (-0.736, 95%CI: -1.355, -0.118, p = 0.020). The other amino acid changes were too low in numbers (n ≤ 2) and thus difficult to interpret.
